# Detection and expression analysis of *tet*(B) in *Streptococcus oralis*

**DOI:** 10.1080/20002297.2019.1643204

**Published:** 2019-07-23

**Authors:** Alexandre Arredondo, Gerard Àlvarez, José Nart, Carolina Mor, Vanessa Blanc, Rubén León

**Affiliations:** a Department of Microbiology, Dentaid Research Center, Cerdanyola del Vallès, Spain; b Departament de Genètica i Microbiologia, Universitat Autònoma de Barcelona, Bellaterra, Spain; c Department of Periodontology, Universitat Internacional de Catalunya, Barcelona, Spain

**Keywords:** Tetracycline, antibiotic resistance, Streptococcus oralis, *tet(B*), horizontal gene transfer, gene expression

## Abstract

Tetracycline resistance can be achieved through *tet* genes, which code for efflux pumps, ribosomal protection proteins and inactivation enzymes. Some of these genes have only been described in either Gram-positive or Gram-negative bacteria. This is the case of *tet*(B), which codes for an efflux pump and, so far, had only been found in Gram-negative bacteria. In this study, *tet*(B) was detected in two clinical *Streptococcus oralis* strains isolated from the gingival sulci of two subjects. In both cases, the gene was completely sequenced, yielding 100% shared identity and coverage with other previously published sequences of *tet*(B). Moreover, we studied the expression of *tet*(B) using RT-qPCR in the isolates grown with and without tetracycline, detecting constitutive expression in only one of the isolates, with no signs of expression in the other one.

This is the first time that the presence and expression of the *tet*(B) gene has been confirmed in Gram-positive bacteria, which highlights the potential of the genus *Streptococcus* to become a reservoir and a disseminator of antibiotic resistance genes in an environment so prone to horizontal gene transfer as is the oral biofilm.

Since their discovery in the 1940s, tetracyclines have been used for many years to treat a wide spectrum of infections. However, resistance to these antibiotics soon appeared, and only the discovery of new derivatives allowed their continued use until the 1980s, when they were gradually replaced by other antibiotics such as fluoroquinolones [1].

Despite their widespread resistance, some tetracyclines are still useful in some treatments, such as in acne vulgaris, periodontitis and infections caused by multidrug resistant (MDR) *Acinetobacter baumannii, Helicobacter pylori* and methicillin-resistant *Staphylococcus aureus* [2–4]. Resistance can occur through efflux pumps, ribosomal protection or enzymatic inactivation. According to http://faculty.washington.edu/marilynr/(last update May 1st, 2019), 60 tetracycline resistance genes have been detected that code for these mechanisms, along with 11 mosaic genes that code for ribosomal protection proteins. Some of these genes, such as *tet*(C) and *tet*(32), have only been found in Gram-positive bacteria, while others such as *tet*(B) have only been found in Gram-negative bacteria. In a recent study by Chander et al., *tet*(B) was found in *Streptococcus suis* isolated from pigs [5], but a lack of full sequencing of the gene prevented the confirmation of such a finding.

In oral biofilm, streptococci are a great reservoir of tetracycline resistance genes [6–8], and one of the most common in the oral environment is *S. oralis* [9]. *S. oralis* is a member of the *Streptococcus mitis* group, known to be a commensal bacterium and opportunistic pathogen [10]. *S. oralis* is an early colonizer of the oral biofilm and plays an important role in its establishment and homeostasis [11]. Moreover, it has been described to antagonize bacteria such as *Streptococcus mutans*, thus preventing the development of dysbiotic biofilm [12]. However, it is one of the most isolated bacteria in endocarditis infections [13,14] and it has been found in numerous types of infection sites [15–19].

Transformation among streptococci is a common event, as they exhibit natural competence and easily acquire foreign DNA. Therefore, the transference of antibiotic resistance genes has been largely studied in this genus [20,21]. Bacteria in the oral environment tend to grow by forming a multispecies biofilm, which increases the chances of horizontal gene transfer and the spread of antimicrobial resistance, creating a large reservoir of antibiotic resistance genes that could be transferred to other bacteria, pathogenic or not [22].

In this study we described the presence of *tet*(B) in two *S. oralis* isolates obtained from subgingival samples of healthy volunteers. Furthermore, in order to prove the presence and transcription of the gene in Gram-positive bacteria, the complete sequence of the gene was obtained, and its expression was evaluated in both isolates.

## Materials and methods

### Sample collection and culture

Subgingival samples were collected from periodontally healthy volunteers in the dental clinic of the Universitat Internacional de Catalunya (UIC), (Barcelona, Spain), and the research protocol was approved by the Ethics Committee of the UIC (study number: PER-ECL-2011–06-NF). Subjects had not received antibiotics 3 months prior to the study and did not show signs of gingivitis or periodontitis. Sterile paper points were inserted into the gingival sulci of teeth 16, 21, 36 and 41 and kept in place for 20 sec. These paper points were pooled in a vial containing 1.5 ml of sterilized reduced transport fluid (RTF) without EDTA [23] and sent to the laboratory at 4ºC for processing within 24 h. Samples were serially diluted and plated on blood agar (Oxoid Nutrient Broth No. 2; Oxoid Ltd, Basingstoke, UK) containing 5% horse blood, hemin (5 mg/l), menadione (1 mg/ml) and 8 µg/ml of tetracycline (Sigma Aldrich, St. Louis, MO, USA) to screen for tetracycline resistant bacteria. Incubation was performed at 37ºC in anaerobic conditions, and after 72 h, all different colony morphologies were subcultured to obtain pure cultures. For each selected isolate, liquid cultures with and without tetracycline (8 µg/ml) were done using modified brain heart infusion (brain heart infusion broth [37 g/l], yeast extract [1 g/l], L-cysteine [0.1 g/l], sodium bicarbonate [2 g/l], and supplemented with hemin [5 mg/l] and glutamic acid [0.25%]). Cultures grew to an OD_600_ of 0.8–1 (≈ 10^8^ cfu/ml), when DNA and RNA extractions were performed.

### Detection of tet(B)

DNA was extracted from all the isolates using the QIAamp DNA Mini Kit (Qiagen, Hilden, Germany) according to the manufacturer’s instructions, quantified using a Nanodrop 2000 UV-vis spectrophotometer (Nanodrop Technologies, Wilmington, DE, USA) and visualized in a 0.5% agarose gel stained with ethidium bromide.

All the isolates were screened for a battery of *tet* genes (data not shown). To detect *tet*(B) the primers tetB-F and tetB-R were used (Table 1) with the following PCR conditions: an initial denaturation of 5 min at 95ºC, 30 cycles of 30 sec of denaturation at 95ºC, 30 sec of annealing at 56.5ºC and 45 sec of extension at 72ºC, and a final extension of 10 min at 72ºC. The result of the PCR was visualized in a 3% agarose gel stained with ethidium bromide, where a band of 242 bp could indicate the presence of *tet*(B).

**Table 1.** List of primers used in this study.

Primers	Sequence 5ʹ – 3’	Product size (bp)	Annealing Temp (°C)	Reference
27-F 1544-R	AGA GTT TGA TCC TGG CTC AG AGA AAG GAG GTG ATC CAG CC	1,533	57	[37]
sodA-F sodA-R	CCI TAY ICI TAY GAY GCI YTI GAR CC ARR TAR TAI GCR TGY TCC CAI ACR TC	500	37	[38]
groEL-F groEL-R	GAH GTN GTI GAA GGI ATG CA ATT TGR CGI AYW GGY TCT TC	800	52	[39]
tetB-F tetB-R	TTG GTT AGG GGC AAG TTT TG GTA ATG GGC CAA TAA CAC CG	242	55	[40]
tetB-insert-F tetB-insert-R	ACC AAA GCT TAG TTA TTC TAC CAC TCC CTA TCA GT CTT CTT CGA ATG CCC TCT TGG GTT ATC AAG	1,319	53	This study
tetB-RT-F tetB-RT-R	TTC AAG TGC GCT TTG GAT GC CGT TGA GAA GCT GAG GTG GT	111	60	This study
16S-So-F 16S-So-R	CGC TCG GGA CCT ACG TAT TA TAC CAG AAA GGG ACG GCT AA	59	60	This study
M1-F M1-NdeI-R	GAT TTT TTA GCA GAA GTA CCG ATA CCA TAT GCC GAT ATT CTA ACC GAA T	629	55	This study
M2-HindIIIF M2-R	CCG ATA CAA GCT TAG TGA CCC GCT TCT GCG A GAC TAT TTG GAC GAC GGG	782	55	This study
ermB-NdeI-F ermB-HindIII-R	GCG TTA GCA TAT GTA CGT TAG ATT AAT TCC TAC CAG CCG ATA CAA GCT TTT ATT TCC TCC CGT TAA ATA	887	55	This study

### DNA sequencing

*tet*(B) was found in two isolates, 444.1 and 469.4, from two different subjects. In order to identify each isolate to the species level, the 16S rRNA*, sodA* and *groEL* genes were amplified using the primers 27-F and 1544-R, sodA-F and sodA-R, and groEL-F and groEL-R, respectively (Table 1). The PCR products were purified using the E.Z.N.A.® Gel extraction Kit (Omega BIO-TEK, Norcross, GA, USA) and sequenced by primer walking at the Genomics and Bioinformatics Service of the Autonomous University of Barcelona (Barcelona, Spain). Primers tetB-insert-F and tetB-insert-R (Table 1) were used to fully sequence the *tet*(B) gene. These primers amplified the gene 41 bp upstream of the start codon and 72 bp downstream of the stop codon. The 1,319 bp amplicons were obtained by PCR and were purified and sequenced by primer walking as described above. The sequences obtained were analyzed using BLASTn software at https://blast.ncbi.nlm.nih.gov/Blast.cgi. Reference sequences for alignment were obtained from the database on http://faculty.washington.edu/marilynr/, specifically the sequences with the accession numbers: J01830, AF223162, V00611, AL513383, AJ277653, AF326777 and AP000342. Additionally, other sequences of *tet*(B) from other species were used to ensure better reliability: CP015434.1, KX458222.1, CP015836.1, NG_048163.1 and LN908249.1. Alignment was performed using the Clustal Omega service at https://www.ebi.ac.uk/Tools/msa/clustalo/.

### RNA isolation and expression of tet(B) gene

RNA was extracted from liquid cultures with and without tetracycline using the High Pure RNA Isolation kit (Roche Diagnostics, Mannheim, Germany) according to the instructions provided by the manufacturer. An extra step to remove any remaining DNA was done using the TURBO DNA-free^TM^ Kit (Thermo Fisher Scientific, Vilnius, Lithuania). The absence of DNA in the samples was verified by PCR using the sets of primers tetB-RT-F/tetB-RT-R, and 16S-So-F/16S-So-R (Table 1), visualizing the results in a 3% agarose gel. The remaining RNA was quantified in a Qubit 4 Fluorometer using the Qubit RNA XR Assay Kit (Thermo Fisher Scientific, Eugene, OR, USA), and its integrity was verified on a 2% agarose gel.

Expression of *tet*(B) was measured by Quantitative Reverse Transcription PCR (RT-qPCR). Reactions were performed with a LightCycler® 480 II Instrument using the LightCycler® 480 RNA Master Hydrolysis Probes kit (Roche Diagnostics, Mannheim Germany), following the instructions provided by the manufacturer. The sets of primers tetB-RT and 16S-So (Table 1) were used together with probes 45 and 66 from the Roche Diagnostics’ Universal ProbeLibrary for the genes *tet*(B) and 16S rRNA, respectively. The RT-qPCR conditions were as follows: i) 75 ng of RNA were used in each reaction for the synthesis of cDNA, which was performed at 63ºC for 3 min; ii) cycling conditions were 95ºC for 30 sec, followed by 45 cycles of 15 sec at 95ºC, 40 sec at 60ºC and 1 sec at 72ºC. Data were analyzed using the LightCycler® 480 Software 1.5. The 16S rRNA gene was used as an endogenous control, and the formula 2^ΔCp*tet*(B)^/2^ΔCp16S rRNA^ was used to evaluate differences in the expression of *tet*(B) in the presence and absence of tetracycline.

### Construction of tet(M) defective mutants

Isolate 469.4 was found to harbor the gene *tet*(M). Therefore, in order to determine the contribution of *tet*(B) to the tetracycline resistance of this isolate, disruption of the gene *tet*(M) was performed by introducing the gene *erm*(B), which confers resistance to erythromycin, between the positions 853 and 1,110 of the *tet*(M) gene. To this end, we made a construct consisting of the gene *erm*(B) surrounded by two sequences homologous to *tet*(M), which we named M1 (the 5ʹ region) and M2 (the 3ʹ region). *erm*(B) was extracted by PCR using the primers ermB-NdeI-F and ermB-HindIII-R from a *S. oralis* clinical strain resistant to erythromycin pertaining to the Dentaid Research Center (Cerdanyola del Vallès, Spain) strain collection. M1 and M2 sequences were obtained by PCR from the isolate 469.4 using the primers M1-F and M1-NdeI-R for the M1 sequence, and M2-HindIII-F and M2-R for the M2 sequence (Table 1). PCR products were purified using the E.Z.N.A.® Gel extraction Kit and digested with *Nde*I and *Hind*III (New England Biolabs Inc., Ipswich, MA, USA). Digested fragments were ligated using T4 ligase (Roche Diagnostics, Mannheim, Germany) according to the manufacturer’s instructions. The construct was then purified using the E.Z.N.A.® Gel extraction Kit and transformed into 469.4 by electroporation using the Gene Pulser Xcell (BioRad, Hercules, CA, USA). Electroporated cells were plated on blood agar plates containing 5 µg/ml of erythromycin (Sigma Aldrich, St. Louis, MO, USA) and incubated in anaerobic conditions for 24–48 h. The recombination event was confirmed by PCR using primers M1-F and M2-R.

### Antibiotic susceptibility testing

Susceptibility to tetracycline was tested in 444.1, 469.4 and 469.4Δ*tet*(M)::*erm*(B)(Ery^R^) using E-test strips (Sigma Aldrich, St. Louis, MO, USA) according to manufacturer’s instructions. *Streptococcus pneumoniae* ATCC 49619 was used as a quality control strain.

## Results

### Species identification and tet(B) sequences

From a library of 448 tetracycline resistant isolates (data not shown), two isolates obtained from two different subjects were found to carry the *tet*(B) gene. Sequencing of the 16S rRNA gene revealed that both isolates were *S. oralis* and shared the same sequence, which showed 100% identity with the 16S rRNA sequences from *S. oralis* ATCC 700233, DSMZ 20,066 and CIP 103216. To further corroborate this result, sequences of the genes *sodA* and *groEL* were also aligned to streptococcal sequences, observing the highest homology with *S. oralis* sequences. To ensure the presence of *tet*(B), full sequencing of the gene and further alignment against reference sequences was performed, showing an identity of 99.99–100% of the *tet*(B) isolated in this study. Sequence J01830 was used as a reference, being the first published sequence of *tet*(B) [24]. Point mutations were found in all of the sequences used compared to J01830 (Supplementary Figure 1). *tet*(B) sequences of isolates 444.1 and 469.4, as well as sequences AF223162, AF326777, AL513383, CP015836.1, KX458222.1, LN908249.1 and NG_048163.1, presented five point mutations in positions 463, 794, 854, 940 and 1012. Sequence CP015434.1 exhibited an additional point mutation in position 1,140, and sequence AP000342 had four of these point mutations in positions 794, 854, 940 and 1,012. All these mutations resulted in amino acid substitutions with the exception of the mutations in position 1,140 (Table 2 and Supplementary Figure 2).

**Table 2.** Positions of nucleotide substitutions in the *tet*(B) sequences used as comparison. Between parentheses, the positions and amino acid substitutions corresponding to each nucleotide substitution. NC: no changes.

	Nucleotide substitution					
Sequences	463A>G (Thr155Ala)	794A>G (Glu265Gly)	854A>T (Asp285Val)	940G>C (Glu314Gln)	1012A>G (Thr338Ala)	1140A>G (NC)
469.4	+	+	+	+	+	
444.1	+	+	+	+	+	
AF223162	+	+	+	+	+	
AF326777	+	+	+	+	+	
AL513383	+	+	+	+	+	
CP015836.1	+	+	+	+	+	
KX458222.1	+	+	+	+	+	
LN908249.1	+	+	+	+	+	
NG_048163.1	+	+	+	+	+	
CP015434.1	+	+	+	+	+	+
AP000342		+	+	+	+	

Sequences obtained in this study were submitted to GenBank (https://www.ncbi.nlm.nih.gov/genbank/), and the following accession numbers were retrieved: MK611935 and MK611934 for the 16S rRNA gene of 444.1 and 469.4, respectively, and MK611936 and MK611937 for the *tet*(B) gene of 444.1 and 469.4, respectively.

### Expression of tet(B)

The level of expression of *tet*(B) in isolates 444.1 and 469.4 was determined comparing log-phase liquid cultures with and without tetracycline of each isolate. The exposure to tetracycline did not decrease the growth rate of the bacteria recovered from the culture and yielded similar amounts of RNA.

**Figure 1.**
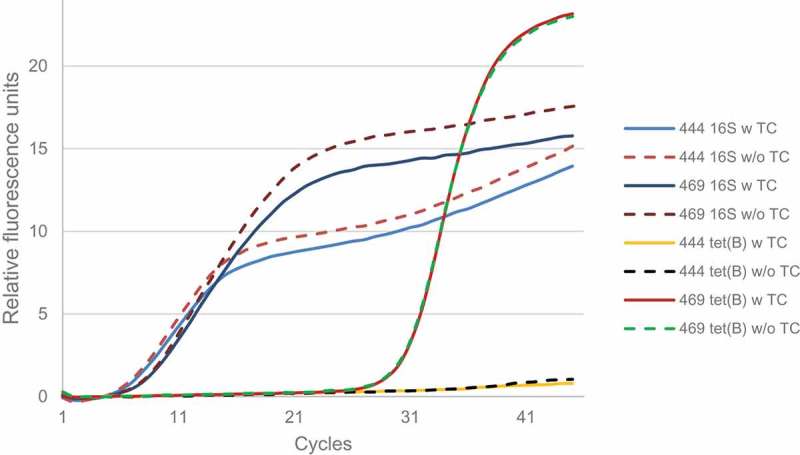
Amplification curves of the 16S rRNA and *tet*(B) genes obtained by RT-qPCR from RNA of 444.1 and 469.4.

A transcriptional analysis of the gene *tet*(B) was performed using RT-qPCR. *tet*(B) mRNA was detected in isolate 469.4 but not in isolate 444.1, despite showing very similar levels of 16S rRNA expression (Figure 1). As for isolate 469.4, no differences were observed in the expression of *tet*(B) when applying tetracycline to the medium. The gene expression value, normalized with the 16S rRNA gene, was 0.06 (log_2_).

According to previous screening for *tet* genes, isolate 469.4 contained a *tet*(M) gene in addition to *tet*(B). In order to determine the influence of *tet*(B) on the tetracycline resistance of the isolate 469.4, the mutant 469.4Δ*tet*(M)::*erm*(B)(Ery^R^) was constructed.

### Antibiotic susceptibility

Susceptibility to tetracycline was tested in isolates 444.1 and 469.4 using E-test strips, observing a minimum inhibitory concentration (MIC) of 32 µg/ml to tetracycline. In order to assess whether tetracycline resistance was being conferred by the gene *tet*(M) as opposed to *tet*(B), susceptibility to tetracycline in the mutant strain 469.4Δ*tet*(M)::*erm*(B)(Ery^R^) was also tested, observing a MIC of 32 µg/ml.

## Discussion

Resistance to tetracyclines is widespread, and although tetracyclines are still useful in some treatments, only the most recent versions are being used to treat multidrug resistant bacteria [25]. Nevertheless, a new formulation of minocycline, a second generation tetracycline, has been proven effective against MDR *A. baumannii* infections, allowing an old antibiotic to be used again [4,25]. Unfortunately, resistance to these antibiotics is possible through different mechanisms such as efflux pumps like the one coded by *tet*(B), which can recognize different tetracyclines as substrates, including minocycline, doxycycline and even glycylcyclines through punctual mutations [26]. *tet*(B) is the most distributed tetracycline resistance gene among Gram-negatives [27] and so far it has not been described in Gram-positives. However, a recent study [5] reported to have found *tet*(B) in *S. suis*, being the first time to describe the presence of this gene in a Gram-positive bacteria. Nonetheless, and although sequencing was performed and 100% identity was obtained, only 54.55% of the gene was covered, raising doubts of whether the gene might or might not be *tet*(B). According to Levy et al. [28], tetracycline resistance determinants should have ≤ 80% of amino acid identity in order to be considered different genes, which makes it impossible for the previously discussed analysis to determine if the gene studied was *tet*(B).

In our study we found two isolates of *S. oralis* susceptible of carrying *tet*(B). In both cases, we performed full sequencing of the gene, resulting in 100% coverage, and compared the sequences to those *tet*(B) sequences previously published, showing some minor differences involving up to six nucleotides in a sequence of 1,206 base pairs, which led to up to five amino acid substitutions in a sequence of 401 amino acids (100% – 98.75% identity). Most of the polymorphisms observed in the *tet*(B) sequences obtained from isolates 444.1 and 469.4 were shared by all the published sequences used in this study with the exception of V00611 and AJ277653, which were identical to the reference sequence J01830 (Table 2). These three sequences were obtained from *Escherichia coli*, although CP015836.1, another *E. coli* sequence used in this study, presented five point mutations compared to J01830, implying that these mutations might have occurred at some point during replication or transference and were maintained in certain strains.

*tet*(B) expression was studied to understand the functionality of the gene in the two *S. oralis* isolates obtained. It is known that, in the presence of tetracycline, the transcription of tetracycline resistance genes is induced [29,30]. For this reason, cultures were made with and without tetracycline and RNA was extracted to quantify the expression of the gene. *tet*(B) mRNA could only be found in isolate 469.4, meaning that the gene was not being expressed under those conditions in isolate 444.1. Furthermore, expression in 469.4 seemed to be constitutive, since its transcription did not depend on the addition of tetracycline, as it was observed once normalized with the expression values of the 16S rRNA gene. Both isolates carried *tet*(M) as well, which might have conferred resistance to tetracycline, as opposed to *tet*(B). We disabled the *tet*(M) gene in the 469.4 isolate through homologous recombination to determine its role in the level of tetracycline resistance, and we found no changes in the level of susceptibility, indicating that *tet*(B) could be responsible for tetracycline resistance in isolate 469.4, although it cannot be ruled out that resistance to tetracycline could be provided by another gene or mechanism.

The presence of *tet*(B) in Gram-positive bacteria is another step in the dissemination of antibiotic resistance genes. Although *tet*(B) seemed to be expressed in only one of the isolates, the presence of this tetracycline resistance gene had never been confirmed in Gram positives. Whether *tet*(B) is being expressed or not, it seems clear that the reservoir for this gene has expanded to the genus *Streptococcus*, an enormous bacterial group, increasing the chances for its dissemination. The transfer of antibiotic resistance genes between Gram-positive and Gram-negative bacteria has been previously reported [31-33], and the oral environment offers a great opportunity for these transfers to occur. The characteristics of the oral biofilm make it prone to genetic exchange, allowing the bacteria that live in such environment to acquire and share antibiotic resistance genes, among others [22,34-36]. Furthermore, the oral environment is a transit area for many bacteria that might end up in other niches of the human body, thus creating the opportunity for these bacteria to acquire genetic determinants present in the oral microbiome. Given that the *tet*(B) sequence analyzed in this study was identical to other sequences of *tet*(B) observed in Gram-negatives, the functionality of this gene, being transferred to Gram-negatives, would be expected. In this study we confirmed the presence and expression of the gene *tet*(B) in Gram-positive bacteria. However, further studies should be conducted in order to establish the function of the gene *tet*(B) in isolates 444.1 and 469.4. Moreover, a full characterization of the surroundings of the gene and its ability to transfer among other streptococci might help to understand the actual potential of the *tet*(B) gene in Gram-positive bacteria.
